# Manejo das Doenças Cardiovasculares em Mulheres: É Trabalho de Todos

**DOI:** 10.36660/abc.20230250

**Published:** 2023-06-02

**Authors:** Gláucia Maria Moraes de Oliveira, Nanette Kass Wenger

**Affiliations:** 1 Universidade Federal do Rio de Janeiro Rio de Janeiro RJ Brasil Universidade Federal do Rio de Janeiro, Rio de Janeiro, RJ – Brasil; 2 Emory University School of Medicine Atlanta Georgia EUA Emory University School of Medicine, Atlanta, Georgia – EUA

**Keywords:** Mulheres, Doenças Cardiovasculares

As doenças cardiovasculares (DCVs) são a principal causa de carga de doença no mundo. Dados recentes do projeto *Global Burden of Disease* (GBD) de 2021 apresentaram valores de 3568,0 DALYs por 100 mil habitantes ( *disability-adjusted life years* , ou anos de vida ajustados por incapacidade; um DALY representa a perda equivalente a um ano de vida saudável) e 162,2 mortes por 100 mil habitantes, com uma prevalência de 6905,6 por 100 mil habitantes no Brasil.^1^ Enquanto progressos consideráveis foram feitos na redução de mortes por DCVs no Brasil de 1980 ao início da década 2020, houve um aumento preocupante na mortalidade bruta e nos DALYs nos últimos anos, principalmente entre as mulheres, devido a infarto do miocárdio, entre outras causas.^2^ Assim, novas estratégias são necessárias para melhorar a saúde cardiovascular.^2 , 3^

O Brasil é um país continental com muitas desigualdades apesar de um sistema de saúde com cobertura universal. O sistema baseia-se, entre outros, no Programa Saúde da Família presente em 5568 cidades de todas as regiões do país. De 1990 a 2019, houve um número maior de mortes e DALYs por acidente vascular cerebral nas mulheres em comparação aos homens em todas as regiões, principalmente Norte e Nordeste, enquanto o número de mortes e DALYs por doença cardíaca isquêmica foi pouco diferente entre os sexos nas regiões.^2^ A Organização para a Cooperação e Desenvolvimento Econômico (OECD) lançou recentemente um relatório sobre atenção primária à saúde no Brasil, que não fez menção à DCV nas mulheres, mas apenas sugestões para a abordagem do câncer de mama e câncer de ovário. No entanto, as DCVs causam duas vezes mais mortes que todas as neoplasias combinadas nas mulheres brasileiras.^4^

De 1990 a 2019, aproximadamente 80% das mortes por DCV no Brasil foram atribuídas aos fatores de risco (FRs). Nesse período, houve uma redução no tabagismo e nos FRs ambientais, mas um aumento nos FRs metabólicos. Pressão arterial elevada e hábitos alimentares não saudáveis são os principais FRs para mortalidade por DCV e DALYs no Brasil.^5^ Enquanto houve uma diminuição na taxa de mortalidade padronizada para a idade atribuída aos FRs avaliados, as taxas brutas de mortalidade aumentaram ou se mantiveram estáveis, exceto em relação ao tabagismo, em ambos os sexos.^5^ Vale ressaltar o risco aumentado de morte atribuída a índice de massa corporal elevado e diabetes nas mulheres.^2^

No estudo Vigitel 2021, uma pesquisa sobre a vigilância de fatores de risco e proteção para doenças crônicas, baseada em entrevistas telefônicas conduzidas em 27 estados do Brasil, a frequência de mulheres obesas foi 22,6%; a frequência da obesidade aumentou até a idade de 64 anos e diminuiu com o aumento da escolaridade, atingindo os valores mais baixos entre as mulheres com 12 ou mais anos de estudo. Ainda, a frequência de atividade física, incluindo tempo de lazer e o tempo equivalente a 150 minutos de exercício moderado, foi maior entre homens (43,1%) que mulheres (31,3%). A atividade física diminuiu com o avanço da idade, e aumentou enormemente com o incremento do nível educacional. Importante ressaltar a frequência dos diagnósticos de diabetes (9,6%) e de depressão (14,7%), ambos mais elevados entre as mulheres.^6^ É fundamental corrigir os hábitos alimentares melhorando o consumo de frutas, verduras, grãos integrais, leite, fibras, cálcio e ácidos graxos poli-insaturados, os quais fazem parte da dieta dos brasileiros, e reduzir o consumo de carne vermelha, carnes processadas, bebidas açucaradas, ácidos graxos trans e sódio, mais consumidos atualmente no Brasil. Ainda, é essencial melhorar os níveis de atividade física neste país continental rico em áreas verdes.^7^

Os FRs específicos do sexo feminino são fundamentais, pois afetam a ocorrência de DCV ao longo da vida de uma mulher. Pré-eclâmpsia, diabetes gestacional, hipertensão induzida por gravidez, parto pré-termo, e fetos pequenos para idade gestacional são indicadores precoces de risco cardiovascular materno.^7^ De acordo com dados do estudo GBD 2019, doenças hipertensivas na gravidez foram a segunda causa de mortalidade e DALYs em mulheres brasileiras em idade reprodutiva.^8^ Dados do estudo CHAP mostraram que o tratamento de hipertensão crônica na gravidez com um alvo de pressão arterial < 140/90 mmHg melhorou os desfechos relacionados com o nascimento do bebê e reduziu a taxa de pré-eclâmpsia com quadros graves.^9^ Interessante mencionar que um estudo que utilizou dados do UK Biobank enfatizou que mulheres com histórico de hipertensão gestacional apresentaram um risco 80% maior de doença coronariana incidente, 70% maior de insuficiência cardíaca incidente, e riscos aumentados de estenose aórtica e regurgitação mitral. Os riscos foram ainda maiores em mulheres que apresentaram hipertensão recorrente durante as gestações. A hipótese dos autores foi que a hipertensão na gestação pode acelerar o envelhecimento cardiovascular nessa população.^10^ Assim, uma história médica detalhada sobre o período da gestação faz-se necessária para melhorar a estratificação dos FRs. Deve ser adicionado os FRs específicos do sexo feminino, que não fazem parte das calculadoras tradicionais de risco cardiovascular, aos FRs tradicionais. Portanto, a gravidez pode ser uma janela para a saúde cardiovascular futura da mulher, e uma parceria com obstetras e ginecologistas torna-se crucial para otimizar a prevenção e o tratamento clínico.^11 , 12^

Existe uma íntima relação entre o Índice Sociodemográfico (SDI) e diminuição de mortes por DCV em mulheres brasileiras. O SDI no Brasil é semelhante ao SDI dos países de alta renda. No entanto, as desigualdades sociais observadas no Brasil são impulsionadores essenciais para DALYs padronizados por idade atribuível a FRs metabólicos e comportamentais de DCV.^2 , 5 , 7 , 8^ Resolver essas desigualdades e os piores resultados associados ao cuidado da saúde cardiovascular das mulheres exigirão investimento em ciência direcionada para o sexo feminino e advocacia para políticas de saúde, bem como incorporar a conscientização do impacto dessas barreiras na prestação de cuidados de saúde para as mulheres.^13^ Assim, são fundamentais as iniciativas para aumentar o conhecimento da importância da saúde cardiovascular no curso de vida das mulheres. Ainda, é essencial melhorar o entendimento da saúde cardiovascular das mulheres em diferentes regiões para serem definidas as políticas públicas e a atenção à saúde específicas, reduzir a desigualdade entre os sexos, e promover a igualdade de gêneros na assistência à saúde das brasileiras.

As sociedades médicas e seus parceiros, tais como organizações não governamentais, são agentes fundamentais para mudar paradigmas e agregar *stakeholders* no manejo das DCVs em mulheres. A cardiologia personalizada, baseada em dados, e centrada no paciente será o futuro da pesquisa clínica. Adicionalmente, esses agentes podem promover a educação médica com base em diretrizes das DCVs e elaborar políticas comunitárias para melhorar a educação pública e traduzir o conhecimento para a prática clínica em benefício das mulheres com DCVs. A incapacidade causada por DCV não diminuiu nos últimos anos, diferentemente do que se observou com a taxa de mortalidade por DCVs. Tal fato é um desafio global importante devido às consequências econômicas e sociais, tais como mortalidade precoce e gastos diretos com saúde. Uma chamada para ação liderada por todos as partes interessadas poderia reduzir a carga global das DCVs, melhorando tanto os FRs modificáveis e os Determinantes Sociais da Saúde (DSS).^7^ Ainda, é necessária uma educação dos profissionais da saúde que seja culturalmente sensível e com a participação da comunidade para prevenção de DCVs nas mulheres. Acesso igualitário à assistência de saúde cardiovascular preventiva baseada em evidências e guiada por diretrizes deveria ser disponível e direcionada a todas as mulheres. Infelizmente, essas diretrizes não são igualmente incorporadas na prática, destacando a necessidade de uma chamadas para ação.^14^

Foram iniciados projetos envolvendo *stakeholders* para melhorar a saúde cardiovascular de mulheres brasileiras. Inicialmente, em 2019, o “Women’s Letter” deliberou ações concretas para reduzir a morbidade e a mortalidade por DCV entre as mulheres. Algumas dessas ações foram: trabalhar coletivamente para a defesa de objetivos globais para prevenir e controlar doenças crônicas não transmissíveis, principalmente DCV no Brasil; estabelecer campanhas de prevenção de DCVs, promover esforços consistentes para alcançar uma meta de redução de 30% na mortalidade por DCVs até 2030; elaborar e sugerir políticas governamentais para fomentar meios favoráveis para reduzir a exposição ao risco, facilitando a adoção de hábitos saudáveis na escola, no trabalho, e ambientes de lazer para combater as DCVs nas mulheres, além de incorporar tecnologias de alto custo efetividade para reduzir morbimortalidade por DCVs nas mulheres.^15^

Desde 2020, e com base nos dados do GBD, vários pesquisadores produziram dados epidemiológicos sobre DCV no Brasil para elucidar disparidades locais e analisar custos associados a internações hospitalares.^2^ No ano passado, foi aprovado o projeto de lei para se comemorar o “Dia Nacional de Conscientização das Doenças Cardiovasculares na Mulher” para enfatizar a necessidade de se assegurar igualdade essencial entre homens e mulheres, particularmente quanto à conscientização sobre as DCVs nas mulheres, doenças negligenciadas no Brasil. Além disso, nós atuamos com a comunidade, a mídia e o governo para aumentar a sensibilização da importância da DCV em geral, principalmente para mulheres. Ainda, por meio do Departamento de Cardiologia da Mulher da Sociedade Brasileira de Cardiologia, foram realizados fóruns com ginecologistas e obstetras para expandir a assistência cardiovascular entre as mulheres e elaborar posicionamentos sobre a saúde cardiovascular das mulheres.^7^

**Figura 1:**
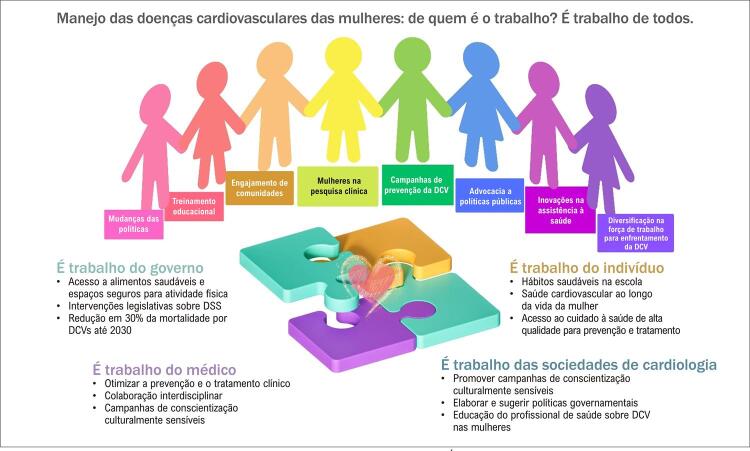
– Manejo das doenças cardiovasculares entre as mulheres: de quem é o trabalho? É de todos. DCV: doença cardiovascular; DSS: Determinantes Sociais da Saúde.

Superar disparidades que afetem a saúde cardiovascular das mulheres inclui mudanças nas políticas, educação e treinamento, inovações na assistência à saúde, e diversificação da força de trabalho em cardiologia.^13^ A chamada para ação do *American Heart Association* “ *Call to Action for Cardiovascular Disease in Women* ” lista uma série de ações para reduzir tais lacunas, entre as quais, destacam-se: otimizar a prevenção e o atendimento médico, promover colaboração interdisciplinar entre cardiologistas, neurologistas, cirurgiões vasculares, angiologistas, médicos de atenção primária, ginecologistas, obstetras e outros profissionais de saúde; promover campanhas de conscientização culturalmente sensível com tradução para as audiências adequadas; engajar comunidades para otimizar a saúde cardiovascular ao longo da vida; direcionar políticas públicas e intervenções legislativas aos DSS, incluindo acesso a alimentos saudáveis e segurança alimentar, espaços seguros para atividade física, ar limpo (ambientes fechados e abertos), e acesso à prevenção e ao tratamento de qualidade.^12^ Assim, o manejo das DCVs das mulheres é trabalho para todos ( Figura 1 ).
