# Supplementary material for: Manejo das Doenças Cardiovasculares em Mulheres: É Trabalho de Todos
Source: Arq Bras Cardiol. 2023 Jun 2;120(5):e20230250. [Article in Portuguese] doi: 10.36660/abc.20230250 (PMC10263394; doi:10.36660/abc.20230250)
Supplement: Translation

# Translation: Managing Women’s Cardiovascular Diseases: It’s Everyone’s Job

**Authors:** Gláucia Maria Moraes de Oliveira, Nanette Kass Wenger

**Affiliations:** 1 Brasil Universidade Federal do Rio de Janeiro, Rio de Janeiro, RJ – Brasil; 2 EUA Emory University School of Medicine, Atlanta, Georgia – EUA

**Keywords:** Women, Cardiovascular Diseases

Cardiovascular diseases (CVDs) remain the leading cause of disease burden in the world. Recent data from the 2021 Global Burden of Diseases project have estimated standardized rates of DALYs of 3568.0 (disability-adjusted life years - one DALY represents the loss of the equivalent of one year of total health) and 162.2 deaths per 100 000 inhabitants, with a standardized prevalence rate of 6905.6 per 100 000 inhabitants, in Brazil.^1^ While considerable progress has been made in decreasing CVD deaths in Brazil from the 1980s to the early 2020s, there has been a worrying increase in crude mortality and DALYs in recent years, especially among young women, due to myocardial infarction, among other causes.^2^ Therefore, new strategies are needed to improve cardiovascular health.^2 , 3^

Brazil is a continental country with many inequalities despite a health system with universal coverage. This system is based on the Family Health Program present in 5568 municipalities across all Brazilian geographic regions. From 1990 to 2019 there was a higher number of deaths and DALYs due to stroke in women versus men in all areas, especially in the North and Northeast of Brazil, while the numbers of deaths and DALYs due to ischemic heart disease were similar in the different regions for both sexes.^2^ The Organization for Economic Cooperation and Development has recently released a report on primary health care in Brazil, with no mention of CVD in women, but only suggestions for approaching breast and ovarian neoplasms. However, CVDs cause twice as many deaths as all neoplasms combined in Brazilian women.^4^

From 1990 to 2019, approximately 80% of the mortality from CVD in Brazil was attributable to risk factors (RFs). In that period, there was a reduction in the smoking and environmental RFs but an increase in the metabolic RFs. High systolic blood pressure and unhealthy dietary habits remain the leading RFs for CVD mortality and DALYs in Brazil.^5^ While there was a decline in age-standardized mortality rates attributable to the assessed RFs, there was stability or an increase in crude mortality rates, except for smoking, in both sexes.^5^ It is worth noting the increased risk of death attributable to high body mass index and diabetes in women.^2^

In the 2021 Vigitel study, a survey on the risk and protective factors of chronic diseases, based on telephone interviews carried out in all 27 Brazilian Federative Units, the frequency of obese women was 22.6% and the frequency of obesity increased up to the age of 64 years and decreased with the increase in schooling, reaching its lowest value among women with 12 and more years of schooling. In addition, the frequency of physical activity, including leisure time and equivalent to 150 minutes of moderate exercise per week, was higher among men than women (43.1% vs 31.3%). Physical activity decreased with increasing age and increased enormously with the level of education. Important to note was the frequency of the medical diagnosis of diabetes (9.6%) and depression (14.7%), both higher among women.^6^ It is crucial to correct the dietary risk by improving the consumption of fruits, vegetables, whole grains, milk, fibers, calcium, and polyunsaturated fatty acids, which are part of Brazilian eating habits, and reducing the consumption of red meat, processed meat, sugar-sweetened beverages, trans-fatty acids, and sodium, which are now highly consumed in Brazil. Furthermore, improving physical activity levels in this continental country with many green areas is essential.^7^

Sex-specific RFs are of fundamental importance and affect the occurrence of CVD throughout a woman’s life. Preeclampsia, gestational diabetes, pregnancy-induced hypertension, preterm delivery, and small for gestational-age newborns are early indicators of maternal cardiovascular risk.^7^ According to data from the GBD 2019 study, hypertensive diseases of pregnancy were the second cause of mortality and DALYs in Brazilian women of childbearing age.^8^ Data from the CHAP trial showed that treating chronic hypertension in pregnancy to a target <140/90 mm Hg improves birth outcomes and reduces the rate of preeclampsia with severe features.^9^ Interestingly, a study using data from UK Biobank has emphasized that women with a history of gestational hypertension had an 80% increased risk for incident coronary heart disease, 70% increased risk of incident heart failure, and increased risks of aortic stenosis and mitral regurgitation. The risks are even higher in women who experience recurrent hypertension during pregnancy. The authors have hypothesized that this syndrome may accelerate cardiovascular aging in this population.^10^ Thus, a detailed pregnancy health history is needed for all women to better stratify RFs, adding sex-specific to traditional RFs, which are not part of the traditional cardiovascular risk calculators. Therefore, pregnancy may be a window to a woman’s future health and partnership with obstetricians and gynecologists is crucial to optimize prevention and clinical care.^11 , 12^

There is a close relationship between the sociodemographic index (SDI) and the decrease in mortality from CVD in Brazilian women. The SDI in Brazil is close to that of high-income countries. However, the social inequalities observed in Brazil are essential drivers for age standardized DALYs attributable to metabolic and behavioral RFs of CVD.^2 , 5 , 7 , 8^ Solving these inequalities and associated worse outcomes in women will require investment in sex-specific science and health policy advocacy, as well as incorporating awareness of the impact of these barriers into our health care delivery.^13^ Therefore, promoting initiatives to enhance knowledge of the importance of cardiovascular health throughout women’s lives is fundamental. In addition, it is essential to better understand the local differences in women’s cardiovascular health to define public policies and health care, reduce the gender gap, and promote sex equity regarding Brazilian health care.

The medical societies and their partners, such as nongovernmental organizations, are critical agents to change paradigms and aggregate multiple stakeholders in managing women’s CVDs. Personalized, data-based, and patient-centered cardiology will be the future of clinical research. In addition, those agents can provide medical education based on the CVD guidelines and build policies for the community to improve public education and translate knowledge into clinical practice to benefit women with CVD. Disability caused by CVD has not declined, differently from that observed in the CVD mortality rate in past years. That disability is a significant global challenge because of its economic and social consequences, such as early mortality and direct health expenditures. A call to action driven by all stakeholders may reduce the burden of CVD globally, improving both modifiable RFs and the social determinants of health (SDOH).^7^ Moreover, culturally sensitive, peer-led community and healthcare professional education is necessary for CVD prevention in women. Equitable access to guideline-approved, evidence-based cardiovascular preventive health care based on available data should be available and targeted to all women. Sadly, these guidelines are not equally incorporated into practice, highlighting the need for a call to action.^14^

We initiated projects involving stakeholders to improve the cardiovascular health of Brazilian women. First, in 2019, the “Women’s Letter” advanced establishing deliberations of concrete actions to reduce CVD morbidity and mortality women. Some of those actions are: to work collectively towards the defense of global goals to prevent and control noncommunicable chronic diseases, especially CVD in Brazil; to establish cardiovascular prevention campaigns, promoting consistent efforts to reach the goal of a 30% reduction in CVD mortality by 2030; to elaborate and suggest governmental policies to foster favorable environments to reduce exposure to risk, facilitating the adoption of healthy habits at school, work, and leisure environments to fight women’s CVD; to work alongside governments to develop and implement a cardiovascular prevention program, in addition to incorporating cost-effective technologies to reduce CVD morbidity and mortality in women.^15^

Since 2020 and based on GBD data, several researchers have built epidemiological data on CVD in Brazil to understand local disparities and analyze costs associated with hospital admissions.^2^ Last year, we passed a “National Day of Awareness of Women’s Cardiovascular Diseases” bill, to emphasize the need to ensure the essential equality between women and men, particularly regarding the awareness about women’s CVDs, which are neglected in Brazil. In addition, we work with the community, the media, and the government to raise awareness of the importance of CVD in general, especially for women. Moreover, through the Women’s Department of the Brazilian Society of Cardiology, we are holding joint forums with gynecologists and obstetricians to expand the reach of women’s cardiovascular care and elaborate position statements on women’s cardiovascular health.^7^

Overcoming disparities that affect the women’s cardiovascular health includes policy changes, education and training, innovations in health care delivery, and diversification of the cardiology workforce.^13^ The American Heart Association’s “Call to Action for Cardiovascular Disease in Women” lists a series of actions to reduce these gaps, of which we highlight: to optimize prevention and clinical care, interdisciplinary collaboration between cardiologists, vascular neurologists, primary care clinicians, obstetricians-gynecologists, and other health professionals; to promote culturally-sensitive awareness campaigns with translation for the appropriate audiences; to engage communities to optimize cardiovascular health across a woman’s life course; to focus the advocacy of public policy and legislative interventions on SDOH, including access to healthy food and food security, safe spaces for physical activity, clean indoor and outdoor air, and access to high-quality care for prevention and treatment.^12^ Thus, managing women’s CVDs is everyone’s job ( Figure 1 ).

**Figure 1:** – Managing women’s cardiovascular diseases: whose job, is it? It´s everyone’s job. CVD: cardiovascular diseases; SDOH: Social Determinants of Health.
